# Recent Developments in the Treatment of Ankle and Subtalar Instability

**DOI:** 10.2174/1874325001711010687

**Published:** 2017-07-31

**Authors:** Kazuya Sugimoto, Shinji Isomoto, Norihiro Samoto, Koujirou Okahashi, Masasuke Araki

**Affiliations:** 1 Department of Orthopaedic Surgery, Nara Prefectural General Medical Center, Nara, Japan; 2 Department of Orthopaedic Surgery, Saiseikai Nara Hospital, Nara, Japan; 3 Developmental Neurobiology Laboratory, Dept. of Biology, Nara Women's Uuniversity, Nara, Japan

**Keywords:** Ankle, Subtalar joint, Instability, Ligament, Repair, Reconstruction, mechanoreceptor, Arthroscopy

## Abstract

It was nearly a centenary ago that severe ankle sprain was recognized as an injury of the ankle ligament(s). With the recent technological advances and tools in imaging and surgical procedures, the management of ankle sprains - including subtalar injuries - has drastically improved. The repair or reconstruction of ankle ligaments is getting more anatomical and less invasive than previously. More specifically, ligamentous reconstruction with tendon graft has been the gold standard in the management of severely damaged ligament, however, it does not reproduce the original ultrastructure of the ankle ligaments. The anatomical ligament structure of a ligament comprises a ligament with enthesis at both ends and the structure should also exhibit proprioceptive function. To date, it remains impossible to reconstruct a functionally intact and anatomical ligament. Cooperation of the regenerative medicine and surgical technology in expected to improve reconstructions of the ankle ligament, however, we need more time to develop a technology in reproducing the ideal ligament complex.

## I ACUTE ANKLE SPRAIN

### 1 Early History of the Ankle Sprain

Ankle sprains without fracture were considered as subtle injuries until the concept of ligamentous injury in the ankle and/ or hindfoot was established. In 1922, Kaufman recognized about rupture of a few ligamentous fiber in the ankle sprain though he described them as minor injuries to periarticular soft tissues [1]. Similarly, in 1933, Dehne pointed out that the most common lesion of a sprained ankle was partial tearing of the ligaments [2], while Elmslie is considered to be the first who reported that the anterior talofibular ligament was commonly involved in ankle sprains [3].

### 2 Classical Imaging of Ankle Instability

The introduction of stress radiography to examine joint instability was first introduced by Bonnin [4]. It was a convenient and objective method to evaluate a ligament rupture other than fractures, and was rapidly accepted among physicians. For a number of years, the gold standard examination for the lateral ankle instability has been talar tilt angle under inversion stress on AP ankle view and anterior translation under anterior drawer test of the talus on lateral ankle view [5]. Stress apparatus was made to reduce variability of the instability recorded on stress radiographs caused by manually applied stress [6]. Instability revealed by stress radiography encouraged surgeons to treat ankle sprains surgically. These latter believed that torn ligaments should be sutured; hence the operative repair of ankle strains was a major treatment until the 1980’s. 

### 3 Controversy in the Treatment of Acute Ankle Sprain

In his review article [7], Kanus examined, the advantages and disadvantages of functional treatment, immobilization in a plaster cast and operative treatment, and he concluded that an operative treatment had no significant advantages over a conservative approach. In fact, higher rates of complications, longer absence period from work and higher costs were associated with the operative treatment. The most common complications of an operative treatment were iatrogenic injuries of the superficial peroneal nerve and or the sural nerve. He reported however that a better ankle stability could be obtained upon operative treatment, hence this was a possible advantage compared with conservative treatment, more particularly in cases of severe sprains with complete tear of 2 or 3 ligaments.

In a randomized controlled trial, Povacz *et al.* [8] reported that there were no differences in clinical outcomes between ankles treated conservatively and those treated operatively. Though, a number of the ankles with fair results were much larger in those with a talar tilt of more than 15 degrees compared with those with a talar tilt of no more than 15 degrees. In 2003, Pinjnenburg *et al.* [9] reported better mechanical stability, fewer recurrent sprains and fewer incidence of residual pain in ankles treated operatively than in those conservatively treated over long follow-up periods. However, controversy still exists on the optimal approaches towards the treatment of acute ankle sprains.

There are 2 major reasons for this controversy. First, it is important to point out that the lateral ankle ligamentous complex consists of 3 ligaments; the anterior talofibular, the calcaneofibular and the posterior talifibular ligament [3]. A given ankle sprain can present different combination of injuries of these ligaments. Hence, it becomes inaccurate to compare the clinical outcome of different combination of ligamentous injuries, and there is limited literature comparing surgical vs. conservative treatments of specific combination of ligamentous injuries.

Most of the acute lateral ankle sprains are isolated anterior talofibular injury or combined injuries of the anterior talofibular and calcaneofibular ligament. A precise diagnosis of the calcaneofibular ligament injury is critical to determine the severity of the lateral ankle sprain.

A second reason resides in the occult subtalar instability accompanying an ankle sprain. The calcaneofibular ligament is not only part of the lateral ankle ligaments, but is also a stabilizer of the subtalar joint. An injury to the calcaneofibular ligament implies an injury to the subtalar joint [10].

Meyer *et al.* [11] first described the subtalar sprain associated with ankle sprain using subtalar arthrography. The presence of subtalar sprain was confirmed in many of the ankle sprain patients included in the study. This explains why the sinus tarsi syndrome [12] commonly occurs in the patient following an ankle sprain, as a result of subtalar ligament injuries such as those affecting the interosseous talocalcaneal and the cervical ligament. However, subtalar symptoms are usually not easy to distinguish from residual symptoms upon the occurrence of an ankle sprain.

### 4 Importance of the Calcaneofibular Ligament Injury

In order to address the controversy over the treatment of acute ankle sprain, a precise assessment of the condition of each individual ligament is essential, especially that of the calcaneofibular ligament.

Stress radiography is the most popular method to assess mechanical instability, and many authors have published criteria of pathological instability [13-16]. The sensitivity and specificity of stress radiography of the ankle is, however, insufficient to assess the degree of injury of individual ligament.

In our study of subtalar arthrography in patients with acute injuries of the calcaneofibular ligament, the accuracy of the method in making a diagnosis of calcaneofibular ligament injury was 76%, compared with 61% for inversion stress radiography and 66% for anterior drawer stress radiography [17]. Using the diagnosis accuracy offered by subtalar arthrography, the results of a conservative treatment were compared between leakage positive and leakage negative series [18]. Only 12% of the patients who showed no leakages had symptoms over a mean follow-up period of 5 years, as opposed to 70% of the patients who showed leakage. We thus concluded that the better approach for patients with acute lateral ankle sprains was the implementation of a week of weight-bearing below the knee cast immobilization followed by 5 weeks of a semi-hard brace, if the injury was exclusively located in the anterior talofibular ligament.

### 5 Non-invasive Examination

Recent developments in MRI and ultrasound allow for the examination of ligaments in a noninvasive manner. Interestingly, however, injuries of the anterior talofibular ligament are predictably detected by MRI and ultrasound, while injuries of the calcaneofibular ligament are not [19, 20].

In our experience of MRI analysis of acute lateral ankle sprains, imaging of the calcaneofibular ligaments was difficult, even in non-injured ankle [21]. In patients presenting combined injuries of the anterior talofibular and calcaneofibular ligaments, the only changes of intensity observed in T2 weighted images were those of the peroneal tendon sheath, suggesting the occurrence of an effusion. Given the cost of the approach and the findings presented so far, MRI was deemed unsuitable for evaluation of the calcaneofibular ligament.

Future advances towards the noninvasive examination of lateral ligaments are based on the use of dynamic ultrasound imaging with high resolution. The examination cost of ultrasound is much lower than that of MRI and only takes a few times to perform. The size of an ultrasound machine is getting smaller hence less space occupying, thus facilitating their use in offices or on the field.

### 6 Future of the Management of Acute Ankle Sprain

In our study, the conservative treatment consisting of a one-week immobilization followed by a functional treatment was determined to be insufficient in addressing the combined injuries of the anterior talofibular and calcaneofibular ligaments [18]. At the moment, we are not in a position to provide any evidence of suitable treatment for patients presenting combined injuries of the anterior talofibular and calcaneofibular ligaments, or for those presenting ruptures of all 3 ligaments. Randomized controlled trials are required to guide the clinical decision making process for patients with combined injuries of the anterior talofibular and calcaneofibular ligaments, and for those with ruptures of all 3 ligaments.

## II CHRONIC OR RECURRENT ANKLE INSTABILITY

### 1 Etiology

Chronic or recurrent ankle instability results from the insufficient healing of the ligaments after an acute sprain or due to deterioration of the ligaments upon repeated sprain, or is also sometimes caused by joint laxity. Muscle strength and proprioception [22] are believed to prevent sprains of the ankle. Various symptoms due to instability, and weakened peroneal muscles and dysfunctions of proprioception worsen instability and increase the risk of recurrent sprains.

Severity of the instability varies widely from an isolated injury of the anterior talofibular ligament to combined injuries. Young active patients may be symptomatic even when the injury is limited in the anterior talofibular ligament, while combined ligamentous injuries in elderly patients may be asymptomatic.

## 2 Conservative Treatment

Peroneal muscles work as pronators of the foot and are believed to protect the ankle from inversion sprains. Training with the goal to increase peroneal muscle strength has been implemented for patients with lateral ankle instability.

**Fig. (1):**
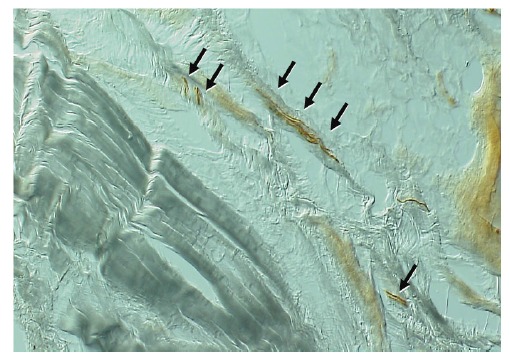
Neurofilament positive fibers observed in the human calcaneo-fibular ligament.

However, if the muscles do not fire rapidly at the moment of inversion sprain, they can

not prevent ankle sprain even if they are sufficiently strong. The reaction of muscles upon stimulation of stretch receptors located in the muscle and in the joint ligaments, was largely investigated; these types of feedback systems are known as proprioception [23]. Mechanoreceptors and nerve networks present around joint capsules are considered as sensors in this kind of system [24, 25]. In preliminary studies, we observed fibers positively bound by anti-neurofilament antibodies in the lateral ankle ligaments (Fig. **1**). Reeducation of the proprioception system is important following an ankle sprain, as this will likely damage the nerve network through rupture of capsules and ligaments.

Balance board training is effective for the prevention of ankle sprain recurrences [26]. Such form of training is believed to result in the reeducation of the nerve network spreading across the ligament and the joint capsules, or result in the improvement of proprioceptions around the ankle.

The effectiveness of external orthosis has been investigated by a number of authors [27-29]. Treatment approaches using braces are convenient and classically represent the first method of choice for chronic or recurrent instability of the ankle. Varied types of braces are manufactured by various companies, hence suitable braces may be found to address various degrees of severity in ankle instability. Nevertheless, some patients will still develop recurrent ankle sprain even if they wear braces.

## 3 Operative Treatment

### a Classical Tenodesis

Operative treatment of the lateral ankle ligament was reported early in the 20^th^ century. Until around 1980, the majority of the reported techniques were tenodesis using the peroneus brevis tendon. The operative procedures described by Elmslie [3], Evan [30], Lee [31], Watson-Jones [32] and Chrisman-Snook [33] represent a variety of tenodesis, which had been adopted by a number of surgeons around the world. The short-term outcome associated with these operations aimed to restore ankle stability, was acceptable.

The main issues resulting from these procedures were their high rate of complications related to nerve injuries due to the width of the skin incision which was necessary to harvest the peroneus brevis tendon [34] and due to restriction of the ankle and hinfoot motion using non-anatomical tenodesis [35]. The sural nerve and/or the superficial peroneal nerve was (were) often affected during the operations causing paeresthesia or hypaesthesia of the lateral and/or dorsal aspect of the foot. Tenodesis using peroneal tendons were gradually replaced by less invasive and more anatomical procedures towards the end of the 20^th^ century.

### b Simple Repair and Reinforcement

Primary repair of the ligaments, as described by Broström *et al.* [36], was a simple method, which did not require sacrifice of the peroneal brevis tendon. This procedure was effective if remnants of the torn ligaments existed, but some authors expressed concerns over the recurrence of ankle instability because of the pathologically vulnerable property of the remnant. In fact, this procedure failed in patients presenting general joint laxity and those with poor remnant. The reinforcement procedure described by Gould *et al.* [37] was one of the solutions brought to address the caveat of Broström’s procedure.

A number of reports have been published on the positive clinical outcomes resulting from the combined use of Broström’s and Gould’s procedures, with low complication rates. One weak point of Gould’s procedure, however, was the restriction of sagittal motion; especially in plantar flexion due to suture of the proximal margin from the extensor retinaculum to the fibular, which sometimes resulted in restriction of hindfoot motion [38].

### c Anatomical Reconstruction

Anatomical reconstructions intended to reproduce original ligament and associated biomechanics were advocated based on studies of detailed local anatomy and biomechanics studies focused on the lateral ligaments of the ankle. Studies performed on cadavers allowed recognition of the overlap of the foot print of the ligaments at fibular attachment and a clearer definition of the isometry of the anterior talofibular and calcaneofibular ligaments [39, 40].

Many anatomical reconstruction techniques consisted in using a tendon graft using an autograft of an allograft [41-46]. Use of an allograft saves operative time and invasion for harvesting the graft, but is not covered by all the types of health insurance. Gracillis tendon, semitendinous tendon, plantarlis tendon and split peroneal tendon are commonly carried out autograft. A few reports of reconstructions using an autograft from the palmaris longs tendon [45] or part of the tendon fascia lata were published.

**Fig. (2):**
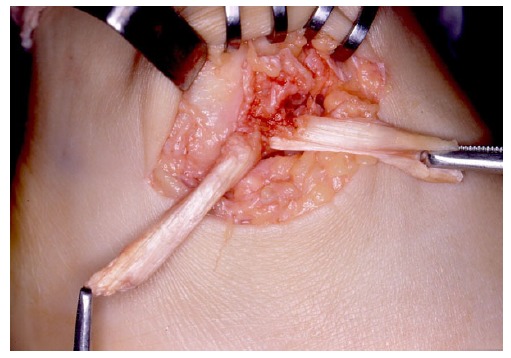
Reconstruction of fibular side enthesis by insertion of the bone part of the bone-patellar tendon autograft isolated from the ipsilatral knee. Each leg of the split tendon is destined to replace the anterior talofibulat ligament (right) and the talocalcaneal ligament (left).

In 13 cases of our series of patients with severe instability, the patellar tendon-bone graft was implemented [44]. The technique described was a unique method to reproduce the structure of the enthesis on the fibular side (Fig. **2**). As public health insurance does not cover the use of allograft in our country, the graft had to be harvested from the patent’s knee. Because of the invasive nature of the procedure to the knee, we stopped using this approach.

In order to circumvent harvesting an autograft from a distant site, reconstructions of the ligament(s) were reported using periosteal flap(s) [47, 48]. The operation had a good indication for young patients who had thick periosteal membrane in the distal fibular, but was difficult to perform in elderly patients with thin periosteum.

### d Artificial Ligament

The use of artificial ligament *i.e.*, Leed-Keio ligament, was reported by Japanese investigators [49]. To our opinion, the procedure still holds a clinical value in patients with general joint laxities. More particularly, Leeds-Keio ligament #10 is fit for the reconstruction of the anterior talofibular with or without the calcaneofibular ligament.

### e Arthroscopic Repair and Reconstruction

Current research trends in relation to the treatment of chronic lateral ankle instability are focused on endoscopic or minimally invasive surgeries. Arthroscopic-assisted reconstruction of combined instability was reported by Lui [50] in 2007. A few years later in 2011, arthroscopic-assisted Broström-Gould for chronic ankle instability was reported by Nery [51]. Arthroscopic surgical interventions attracted the attention of foot & ankle surgeons, as well as arthroscopic surgeons, and also led to the development of innovative suture devices and suture anchors. In the early series, suture of the extensor retinaculum to the fibular was performed and the operation kit was called ArthroBroström^®^, though the technique resembled Gould’s augmentation procedure.

In 2013, all -inside arthroscopic repair of the anterior talofibular ligament was reported by Vega *et al.* [52], and was based on the use of knotless anchor suture. The technique introduced was a simple repair of the ligament itself. This technique seemed more anatomical than the procedure involving the suture of the retinaculum to the fibula.

The strength and stiffness of the arthroscopic Broström repair were more favorable in comparison to an open repair procedure, and was more feasible in clinical settings, as suggested by fresh-frozen cadaver study [53]. If the position of the anchor and suture of the ligament or retinaculum were correctly placed, the biomechanical properties were considered to be the same as those resulting from an open surgery. It is thus essential to establish a simple and easy technique to reduce technical errors in the early phase of the learning curve.

Arthroscopic repair techniques are primarily aimed to repair the anterior talofibular ligament, and fail in the repair of the calcaneofibular ligment. Arthroscopic reconstruction of the lateral ligaments using the gracilis tendon has been proposed by the ankle instability group [54]. The procedure may be performed percutaneously without arthroscopic control. Minimally invasive treatments are attractive to patients suffering from chronic ankle instability regardless of whether the procedures are arthroscopic or not.

Of 6 recent arthroscopic repair studies, 31 complications were reported amongst a total of 178 operations (17.4%) [55]. Arthroscopic repair or reconstruction of the lateral ankle ligaments have just started to enter clinical settings. There is a need for further development in operative techniques, devices and preoperative evaluation of ligament conditions in order to promote the use of minimally invasive management approaches for ankle instability.

## 4 Subtalar Instability

Isolated subtalar instability without ankle instability is a rare condition. The interosseous talocalcaneal ligament is a strong ligament and it seldom ruptures without injuries to the ankle. As mentioned above in relation to acute sprains, the calcanefibular ligament is a stabilizer of the subtalar joint. Many patients with dysfunction of the calcanefibular ligament have potential injuries to the interosseous talocalcaneal ligament [56].

The sinus tarsi syndrome is known as a unique clinical disorder, which develops subsequently to an “ankle” sprain. The clinical features of the syndrome are pain in the sinus tarsi and unstable feeling of the hindfoot without mechanical instability of the ankle [12, 57]. Absence of mechanical instability of the ankle does not rule out a history of ankle sprain. It appears that the sinus tarsi syndrome is a residual symptom of the partial tear of the interosseous talocalcaneal ligament or that of the cervical ligament accompanying the ankle sprain.

In subtalar arthrography studies of the chronic ankle sprain, we found that leakage of the contrast medium from the posterior facet of the subtalar joint to the ankle joint and/or to the peroneal tendon sheaths involved a tear of the calcaneofibular ligament [56]. These studies suggested that chronic ankle instability involving the calcaneofibular ligament were also associated with injuries of the subtalar joints.

In our hands, the volume of the interosseous talocalcaneal ligament or the cervical ligament was decreased and spaces are occupied with synovial tissue and or fibrous tissue. Those findings are supported by literatures [58, 59]. Arthroscopic debridement of those tissues was effective for the syndrome, we thus suspect that the partial tear of the interosseous talocalcaneal ligament or the cervical ligament is the most likely underlying cause of the syndrome.

## 5 Future Developments in the Clinical Management of Chronic or Recurrent Instability of the Ankle and Hhindfoot

The design of reconstruction procedures in patients presenting ankle instability has become more and more anatomical and less invasive than previously. Tendon graft remains the gold standard, however, it fails to reproduce the original structure of the ankle ligament complex constituted of a ligament, enthesis at both ends and proprioceptive functions. Currently, there are no methods available to reconstruct functionally intact and anatomical ligaments of the ankle.

Recent development in regenerative medicine is expected to bring a breakthrough in the traumatology of the joint. Biocompatible materials mimicking the structure of the bone, cartilage and collagen fiber are nearing the stage of clinical applications. Simple structures of the bone, cartilage and collagen fiber are in the stage of possible clinical use. However, further investigations are required to reproduce the anatomical ligament complex. Cooperation of the regenerative medicine and surgical technology in expected to improve reconstructions of the ankle ligament, if they are not ideal yet [60].

## III ANKLE INSTABILITY AND OSTEOARTHRITIS

Harrington [61] first reported that prolonged instability of the ankle may be associated with osteoarthritis of the ankle. Since then, many physicians have accepted chronic ankle instability as a cause of osteoarthritis. However, the number of patients suffering from osteoarthritis of the ankle is much smaller than that of the knee when considering a large group of patients with ankle sprain.

We suspected that risk factors, other than ankle instability, could explain the occurrence of ankle osteoarthritis, and we searched for the background of patients presenting a chronic instability of the ankle with chondral damages. One study reported that other risk factors associated with ankle osteoarthritis were higher age and medial inclination of the ankle plafond, as detected by inversion stress radiography [62].

Low tibial or distal tibial oblique osteotomies are indicated for patients presenting stage III osteoarthritis of the ankle [63, 64]. To protect the ankle from the end stages of osteoarthritis, it is essential to repair or reconstruct the ligaments in the early stage of osteoarthritis, and to correct the inclination of the plafond in the later stages.

## CONCLUSION

The anatomical ligament structure of a ligament comprises a ligament with entheses at both ends and structure should also exhibit proprioceptive function. To date, it remains impossible to reconstruct a fuctionally intact and anatomical ligament. Cooperation ofthe regenerative medicine and surgical technoligy in expected to improve reconstructions ofthe ankle ligament, however, we need more time to develop a technology in reproducing the ideal ligament comlplex.
